# Classification of Incomplete Data Based on Evidence Theory and an Extreme Learning Machine in Wireless Sensor Networks

**DOI:** 10.3390/s18041046

**Published:** 2018-03-30

**Authors:** Yang Zhang, Yun Liu, Han-Chieh Chao, Zhenjiang Zhang, Zhiyuan Zhang

**Affiliations:** 1 Key Laboratory of Communication and Information Systems, Beijing Municipal Commission of Education, School of Electronic and Information Engineering, Beijing Jiaotong University, Beijing 100044, China; 14111004@bjtu.edu.cn (Y.Z.); 13111005@bjtu.edu.cn (Z.Z.); 2 School of Information Science and Engineering, Fujian University of Technology, Fuzhou 350118, China; hcc@mail.ndhu.edu.tw; 3 School of Mathematics and Computer Science, Wuhan Polytechnic University, Wuhan 430024, China; 4 Department of Electrical Engineering, National Dong Hwa University, Hualien 97401, Taiwan; 5 Department of Computer Science and Information Engineering, National Ilan University, Yilan 26047, Taiwan; 6 School of Software Engineering, Beijing Jiaotong University, Beijing 100044, China; zhangzhenjiang@bjtu.edu.cn

**Keywords:** classification, incomplete data, evidence theory, extreme learning machine, wireless sensor network

## Abstract

In wireless sensor networks, the classification of incomplete data reported by sensor nodes is an open issue because it is difficult to accurately estimate the missing values. In many cases, the misclassification is unacceptable considering that it probably brings catastrophic damages to the data users. In this paper, a novel classification approach of incomplete data is proposed to reduce the misclassification errors. This method uses the regularized extreme learning machine to estimate the potential values of missing data at first, and then it converts the estimations into multiple classification results on the basis of the distance between interval numbers. Finally, an evidential reasoning rule is adopted to fuse these classification results. The final decision is made according to the combined basic belief assignment. The experimental results show that this method has better performance than other traditional classification methods of incomplete data.

## 1. Introduction

Recently, the Dempster–Shafer evidence theory (DST) has attracted extensive attention for its advantages in combining information from distinct sources into a unified source [1,2]. In wireless sensor networks (WSNs), evidence theory provides a flexible solution to decrease the uncertainty and imprecision of decisions when dealing with the data provided by sensors [3], and it has been widely used in many complex applications, such as object classification, environmental monitoring, disaster search, and information fusion [4,5]. In this paper, we mainly focus on how to classify the incomplete data collected from several sensor nodes in WSNs. Considering the influence of limited node energy and complicated application surroundings, incomplete data, i.e., the missing attribute values, are often reported by sensor nodes [6]. In the process of classifying the incomplete data, evidence theory plays a crucial role. In most cases, evidential reasoning can efficiently reduce the uncertainty and imprecision caused by missing values based on the known values of the attributes. Furthermore, many new schemes have been proposed in recent years to improve the accuracy of the classification of incomplete data. In [7], the nature of missing values was discussed, and three different types of missing data were introduced in [8,9], as follows:

1. Data missing completely at random (MCAR): If a missing variable is independent of the value of itself and any other variable, then it can be denoted as MCAR.
2. Data missing at random (MAR): If a missing value does not depend on the missing variable and it may be related to the known values of other variables, then the missing data can be denoted as MAR.
3. Data not missing at random (NMAR): If a missing value depends on the missing variable itself, then the missing data is denoted as NMAR.

Most current mechanisms for processing the missing data were designed based on the assumptions of MCAR and MAR, and a possible method for dealing with NMAR is searching the model that can characterize the missing data. In most cases, it is of great difficulty to obtain an appropriate model for probability distribution of the missing data [10]. Consequently, in this work, we mainly focus on dealing with MCAR or MAR data in WSNs.

Recently, a great number of methods have been proposed to solve the above two problems. The simplest way is to ignore the unknown attribute values, and take only the known values into consideration. This method is acceptable when the missing values take a small portion of the whole data set. Dempster et al. introduced the expectation maximization (EM) algorithm [11] to estimate the values of missing data by employing the maximum likelihood (ML). The EM algorithm outperforms the method that ignores the unknown values; however, it always yields a lower convergence speed. Subsequently, many estimation (imputation) approaches have been proposed to fill the missing data on the basis of statistical and machine-learning methods, such as mean imputation (MI) [12], K-nearest neighbor imputation (KNNI) [13], regress imputation [7], multiple imputation [14], multiple imputation by chained equations (MICE) [15], singular value decomposition (SVD) [16], fuzzy K-means (FKM) imputation (FKMI) [17], support vector regression imputation (SVRI) [18], extreme learning machine imputation (ELMI) [8,19]. The SVR is the support vector machine (SVM) version for regression, and an estimation of the missing values is achieved by minimizing the observed training error. In FKMI, the missing values are filled in terms of the clustering centers and the distances between the observations and these centers. In MICE, each variable with a missing value is modeled conditionally according to the other variables in the data. Though multiple imputation combines several distinct imputations into an over-estimate, it is not suitable for WSNs considering the high energy consumption. In MI, the missing values are estimated based on the mean of the known values in the same dimensions. However, the estimations of the missing values generated by MI may be imprecise. Sometimes, the missing values in different classes are likely to be filled with the same estimates. In KNNI, the missing values are replaced by the attribute values of the K-nearest complete neighbors, and it is a precise and efficient imputation method. However, compared with other methods, KNNI has a relatively higher computation complexity and, hence, if this method is applied in WSNs, the lifetime of the network will be shortened greatly. In ELMI, the missing values are estimated by the output of the ELM network [20]. ELM networks usually offer a good solution to address the missing value problems even if half of the training data are missing [8]. In addition, because the input weights of ELM are generated randomly, ELMI can provide a good performance in solving the missing data problems with an extremely fast learning speed. As a result, in this paper, the ELMI is applied to predict the missing values of incomplete data. Firstly, the missing values of incomplete data are filled with ELMI, and then the proper evidential reasoning method will be used to classify the data with the estimated values.

**Figure 1:**
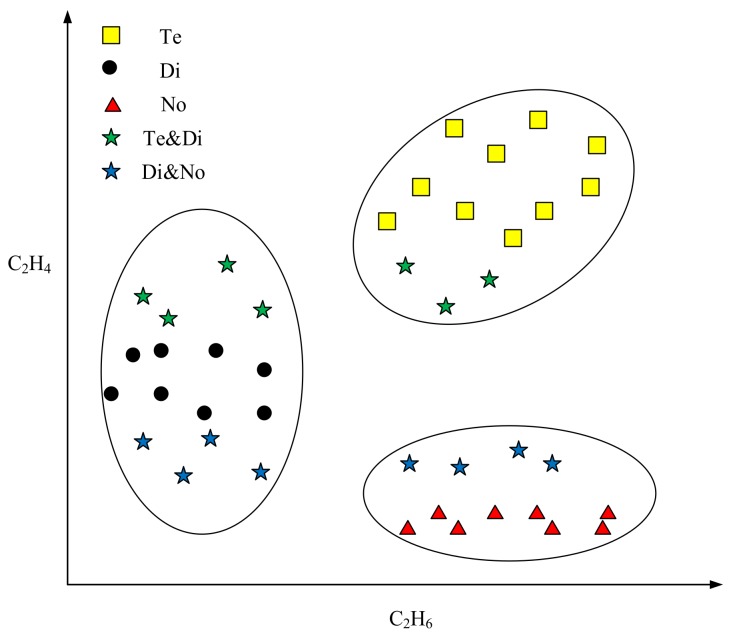
The presentation of three states of the transformer. No: normal state; Te: temperature fault; Di: discharge fault.

In most classification tasks, the object is classified into the class with the largest probability. However, in fact, it is hard to commit an object with incomplete data to a precise class [6]. Considering the uncertainty of the incomplete data, each missing value of the data may receive several possible estimations. Thus, different estimated values may induce various classification results. Figure 1 shows a transformer state diagnosis example. There are several gases in the transformer’s inner space, such as H_2_, CH_4_, C_2_H_6_, and C_2_H. For convenience, we use C_2_H_6_ and C_2_H_4_ to decide the state of the transformer. In Figure 1, the horizontal axis represents the percentage of C_2_H_6_, and the vertical axis denotes the percentage of C_2_H_4_. There are three types of states of transformer, including normal state (No), temperature fault (Te), and discharge fault (Di). When the faults appear, the percentage of each gas will change. We assume that all the values of C_2_H_6_ are missing, and only the C_2_H_4_ values are available. In such a case, by observing the C_2_H_4_ values alone, it is hard to classify the green stars into the proper state. If the estimations of C_2_H_6_ values of green stars are large, then they are likely to be assigned to the fault state Te. However, if the estimations of C_2_H_6_ values of green stars are small, then they are likely to be classified to the state of Di. The identification process of blue stars is similar to that of identifying the green stars, and it is impossible to distinguish the states of blue stars in terms of the C_2_H_4_ values alone. The reason for such imprecise and uncertain classifications is that the known information is insufficient to make precise classifications.

To deal with such uncertain and imprecise estimated values, evidence theory is used in this work. We assume that a missing attribute may have several possible estimated values, and the mass function of an evidential reasoning framework can be used to express the uncertainty of these estimated values very well [21].

The Dempster–Shafer evidence theory [22], which is also called evidential reasoning or the belief functions theory, has been proved to be valuable as a solution for dealing with uncertain and imprecise information [23], and it has been widely applied in many applications, such as state estimation [24], target recognition [2], data classification [25], and information fusion [26]. On the basis of DST, a number of fusion rules have been proposed [27]. In [28], the evidential K-nearest neighbors classification scheme was introduced. Then, the evidential neural network classification scheme was proposed by Denoeux in [29]. In [30], Liu et al. innovatively developed the credal classification rule (CCR). By using CCR, an object with imprecise values of attributes is classified into the compound class that is equal to the disjunction of several classes. In [10], the effectiveness of credal classification for incomplete data was demonstrated, and the CCR is adequate to combine the uncertain and imprecise estimations of the missing value.

Considering the limitations of the energy and calculation capacity of sensor nodes, this paper proposes a novel classification method for incomplete data in WSNs. This method uses the simple and efficient regularized ELM to estimate the missing values of incomplete data, and combines the uncertain and imprecise estimations on the basis of evidential reasoning, which has a lower error rate. In addition, a new basic belief assignment (BBA) construction strategy based on interval numbers is also introduced. The proposed classification method can obviously reduce the negative effect of missing values on the classification results and captures a reasonable decision according to the observations of sensor nodes.

The rest of this paper is organized as follows. Section 2 briefly introduces the basics of evidence theory and ELM. The proposed imputation method and evidence combination approach are presented in Section 3. Section 4 provides the experimental results to demonstrate the performance of our method. Finally, this paper is concluded in Section 5.

## 2. Preliminary Work

In this section, some basic concepts of belief function theory and ELM are introduced.

### 2.1. Basis of Belief Function Theory

The Dempster–Shafer evidence theory (DST) introduced by Shafer is also known as belief function theory [22]. In this theory, the frame of discernment Ω is a finite set, whose elements are exhaustive and mutually exclusive, and it is denoted as $\Omega =\{w_{1},w_{2},\ldots ,w_{i},\ldots ,w_{c}\}$. $2^{\Omega }$ is the power set of the frame of discernment, which represents the set of all possible subsets of Ω, indicated by $2^{\Omega }=\left\{\phi ,\left\{w_{1}\right\},\ldots ,\left\{w_{n}\right\},\left\{w_{1},w_{2}\right\},\ldots ,\left\{w_{1},w_{2},\ldots w_{i}\right\},\ldots ,\Omega \right\}.$ Given an object X, it can be classified as any singleton element and any sets of elements in $2^{\Omega }$ with a basic belief assignment (BBA). The BBA is also known as the mass function, which is a mapping $m:\;2^{\Omega }\to \left[0,1\right]$ satisfying $\sum_{A\in 2^{\Omega }}m\left(A\right)=1$, m(ϕ)=0. The function m(A) is used to quantify the degree of belief that is exactly assigned to the subsets A of Ω. If m(A)>0, the subset A can be called the focal elements of the mass function m(⋅). The mass values assigned to compound elements can reflect the imprecise observation of object X.

The mass function m(⋅) is always associated with three main functions, including the belief function Bel(⋅), the plausibility function Pl(⋅) and the pignistic probability function BetP(⋅), which are defined as follows, respectively:

(1) $$Bel(B)=\sum_{A\subseteq B}m\left(A\right)$$

(2) $$Pl(B)=\sum_{A\cap B\neq \emptyset }m\left(A\right)$$

(3) $$BetP\left(w\right)=\sum_{w\in A,A\subseteq \Omega }\frac{1}{\left|A\right|}m\left(A\right)$$

where m(⋅) is the focal elements on Ω, and |A| denotes the cardinality of focal elements A. All three functions can be employed to make a decision on an unknown object according to a few rules, such as selecting the class with maximum BetP.

Assuming that there are two pieces of evidence denoted by $m_{1}$ and $m_{2}$, the popular Dempster’s combination rule can be used to combine them as follows:

(4) $$\begin{array}{l} m_{⊕}\left(A\right)=m_{1}\left(B\right)⊕m_{2}\left(C\right)=\\ \left\{ \begin{array}{ll} 0, & B\cap C=\phi ;\\ \frac{\sum_{B\cap C=A,\forall B,C\subseteq \Omega }m_{1}\left(B\right)\times m_{2}\left(C\right)}{1-\sum_{B\cap C=\phi ,\forall B,C\subseteq \Omega }m_{1}\left(B\right)\times m_{2}\left(C\right)}, & B\cap C\neq \phi , \end{array}\right. \end{array}$$

where $\sum_{B\cap C=\phi ,\forall B,C\subseteq \Omega }m_{1}\left(B\right)\times m_{2}\left(C\right)$ represents the conflict between $m_{1}$ and $m_{2}$, which is used to redistribute the conflicting mass values. Dempster’s combination rule is commutative and associative. It provides a simple and flexible solution for data fusion problems. However, in some conflicting cases, Dempster’s combination rule often has a poor performance and results in unreasonable results. Consequently, a series of alternative combination methods has been proposed in recent years, such as the conjunctive combination rule, the disjunctive combination rule, and Yager’s combination rule [31,32]. Most of these methods can provide reasonable solutions by redistributing the conflicting beliefs to corresponding focal elements. In this work, in order to reveal the imprecision of classification caused by missing values, a flexible combination method that conditionally transfers the conflicting beliefs to selected compound classes based on the original Dempster’s rule is introduced in Section 3.2.

### 2.2. Overview of Extreme Learing Machine

**Figure 2:**
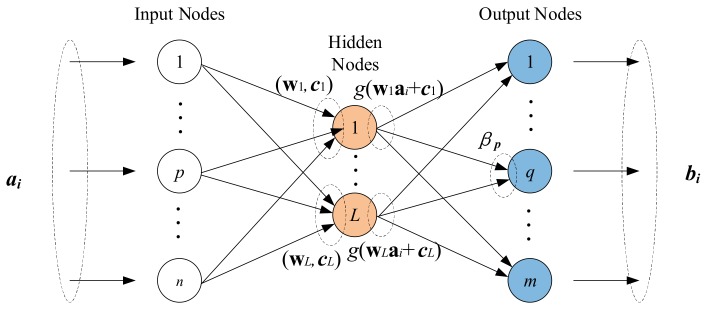
The schematic diagram of extreme learning machine (ELM).

ELM, proposed by Huang et al. in [33], is a type of single-hidden layer feedforward neural network (SLFN). In ELM, the input weights of the hidden neurons are randomly assigned without any iterative computation, and the output weights are calculated by the Moore–Penrose generalized inverse of a matrix. Compared with other traditional SLFNs based on gradient-descent learning algorithms, the ELM offers a faster training speed and better generalization performance [34]. The schematic diagram of ELM is shown in Figure 2.

As shown in Figure 2, these is a set of N observations $\left(a_{i},b_{i}\right),i\leq N$, where $a_{i}={\left[a_{i1},a_{i2},\ldots ,a_{in}\right]}^{T}\in R^{n}$ is the input data and $b_{i}={\left[b_{i1},b_{i2},\ldots ,b_{im}\right]}^{T}\in R^{m}$ is the output data. If there are L hidden layer nodes, then the output of the network can be expressed in the following form:

(5) $$b_{i}=\sum_{j=1}^{L}\beta_{j}g\left(w_{j}a_{i}+c_{j}\right),\;i=1,\ldots ,N$$

where $w_{j}={\left[w_{j1},w_{j2},\ldots ,w_{jn}\right]}^{T}$ is the input weight between the *j*-th hidden node and the input node $a_{i}$, $c_{j}$ is the bias of the *j*-th hidden node, $\beta_{j}={\left[\beta_{j1},\beta_{j2},\ldots ,\beta_{jm}\right]}^{T}$ is the output weight between the *j*-th hidden node and the output node $b_{i}$, and g is an activation function. Equation (5) can be expressed in the matrix form as:

(6) Hβ=B

where

(7) $$H={\left[\begin{array}{ccc} g\left(w_{1}a_{1}+c_{1}\right) & \cdots & g\left(w_{L}a_{1}+c_{L}\right)\\ \vdots & \cdots & \vdots \\ g\left(w_{1}a_{N}+c_{1}\right) & \cdots & g\left(w_{L}a_{N}+c_{L}\right) \end{array}\right]}_{N\times L},\;\beta ={\left[\begin{array}{c} \beta_{1}^{T}\\ \vdots \\ \beta_{L}^{T} \end{array}\right]}_{L\times m}\mathrm{and}\;B={\left[\begin{array}{c} b_{1}^{T}\\ \vdots \\ b_{N}^{T} \end{array}\right]}_{N\times m}.$$

As aforementioned, the ELM randomly initializes the parameters $\beta_{j}$ and $c_{j}$. The output weights are computed by the Moore–Penrose generalized inverse of matrix H, which has the following form:

(8) $$\beta =H^{†}B.$$

The original ELM is designed based on the empirical risk minimization (ERM) principle, and the output weight $\beta =H^{†}B$ is calculated by the minimum norm least squares method. In terms of statistical learning theory, if the training samples are limited, then ERM is likely to bring a risk of overfitting. To avoid the problem of overfitting, a regularized ELM, which uses the tradeoff between empirical risk and structural risk to reflect the real risk of learning, was proposed in [35]. In regularized ELM, the empirical risk is defined by ${\left\|\varepsilon \right\|}^{2}$, and the structural risk is defined by ${\left\|\beta \right\|}^{2}$.

Thus, according to Equation (6), the model of the regularized ELM can be formulated as:

(9) $$\begin{array}{l} \min\frac{1}{2}{\left\|\beta \right\|}^{2}+\frac{1}{2}r{\left\|\varepsilon \right\|}^{2}\\ s.t.\sum_{j=1}^{L}\beta_{j}g(w_{j}a_{i}+c_{j})-b_{i}=\varepsilon_{i},\;i=1,\ldots ,N \end{array}$$

where $\varepsilon =\left[\varepsilon_{1},\varepsilon_{2},\ldots ,\varepsilon_{N}\right]$ and the parameter r are used to adjust the proportion between ${\left\|\varepsilon \right\|}^{2}$ and ${\left\|\beta \right\|}^{2}$. In [35], the value of r is recommended to be selected from the set $\left[2^{-50},2^{-49},\ldots ,2^{50}\right]$ in terms of various tests. Then, the output weight β can be calculated by:

(10) $$\beta ={\left(\frac{1}{\gamma }+H^{T}H\right)}^{†}H^{T}B.$$

ELM can provide a better universal approximation capability and generalization ability, and has been widely used in many fields (e.g., classification, regression, and unsupervised learning) [34]. As a result, to deal with the incomplete data in WSNs, the extreme learning machine imputation (ELMI) will be discussed in the next section.

## 3. Classification of Incomplete Data with ELMI and DST

### 3.1. Extreme Learning Machine Imputation

The regularized ELM can provide a better generalization performance than that of ELM. At the same time, the training time of the regularized ELM is similar to that of ELM. Since incomplete data are very common and pervasive in classification problems, the regularized ELM is adopted here to estimate missing attribute values.

Let us consider a *c*-class problem, where the object may belong to *c* different classes. There are *n* training samples with complete data $\;X=\{x_{1},\ldots ,x_{n}\}$. Each training sample has k attributes. We split the training samples into *c* different training classes $T_{s},s=1,\ldots ,c$ in terms of their class label at first. In this way, *c* training classes can be generated. The dimensions of missing attributes of the monitoring object are marked as a set K={1,…,k} where K can be a single attribute or multiple attributes. In each training class $T_{s}$, the values of attributes K are selected as output data, and the values of the rest of the attributes are selected as input data to compute matrix $H_{s},s=1,\ldots ,c$ according to Equation (7). Then, if there are L hidden layer nodes, *c* output weight vectors $\beta_{s}={\left[\beta_{s1},\beta_{i2},\ldots ,\beta_{iL}\right]}^{T},s=1,\ldots ,c$ are calculated according to Equation (10). Lastly, the known attribute values of the monitoring object are selected as input data, and the output data can be calculated to estimate the missing values according to Equation (6). By using *c* different $\beta_{s}$, *c* possible estimations of each missing attribute are computed.

The aforementioned ELMI method can be performed in four steps:

1. Divide the training samples into *c* different training classes $T_{s},s=1,\ldots ,c$ according to the class label of the samples;
2. Determine the missing attributes K of the monitoring object, K={1,…,k};
3. Select the values of attributes K of each training class as the output data, and select the rest of the attribute values of each training class as input data to compute *c* output weight vectors $\beta_{s}={\left[\beta_{s1},\beta_{i2},\ldots ,x_{iL}\right]}^{T},s=1,\ldots ,c$ using Equation (10);
4. Select the known attribute values of the monitoring object as input data, and calculate the output data to estimate the missing values according to Equation (6).

For *m* monitoring objects $\;Y=\{y_{1},\ldots ,y_{m}\}$ with incomplete data, we can get *c* versions of estimations of the missing attributes by using ELMI, and the missing values are filled with each version of estimations. As a result, an object $y_{j},j=1,\ldots ,m$ can have *c* filled attribute vectors $y_{j}^{s}=[y_{j1}^{s},\ldots ,y_{jk}^{s}],s=1,\ldots ,c$.

The edited vectors $y_{j}^{s}$ are derived from *c* different training classes. The edited attribute values are most likely to be imprecise and mutually conflicting. In addition, by looking at these attribute values alone, it is impossible to find the real class of the object $y_{j}$. To classify the object with estimated values, a classification method based on evidential reasoning is proposed in Section 3.2.

### 3.2. The Classification Method Based on Evidence Theory

Considering the uncertainty and imprecision of attribute vectors $y_{j}^{s},s=1,\ldots ,c$, DST is adopted to classify the objects. DST uses the frame of discernment Ω to model all possible classes of an object, and uses the BBA to represent the degree of support of each class. In DST, the compound classes (disjunction of several singleton classes) are allowed to reflect the imprecision of the object class. Different BBAs characterize the uncertainty of the object class. Since the ELMI provides multiple attribute vectors, the decision on an object class should be made by fusing these vectors. In most cases, Dempster’s combination rule offers an effective solution for multi-sensor data fusion. However, when the BBAs from two pieces of evidence are in conflict, a counterintuitive result is often produced by the classical Dempster’s combination rule. Considering that the multiple attribute vectors derived from diffident estimations are probably mutually conflicting, it is necessary to find a rational combination method to fuse them. In [10], Liu et al. introduced a flexible solution that transfers the conflicting beliefs to selected compound classes. The credal classification method is also used to reduce the misclassification errors. In this paper, inspired by this idea, we propose a novel classification method based on evidential reasoning.

#### 3.2.1. The Classification Based on Interval Numbers and DST

As mentioned in Section 3.1, the ELMI provides *c* versions of estimations, and *c* edited attribute vectors are produced. For each attribute vector $y_{j}^{s},s=1,\ldots ,c$, any standard classifier can be used to generate a classification result. Under the evidence theory framework, this result should be a normal BBA. Therefore, a simple classifier based on DST is presented here.

In a *c*-class problem, there are *n* training samples $X=\{x_{1},\ldots ,x_{n}\}\;$, and *m* test samples $\;Y=\{y_{1},\ldots ,y_{m}\}$ on the frame of discernment $\Omega =\{w_{1},w_{2},\ldots ,w_{i},\ldots ,w_{c}\}$. For each test sample $y_{j},j=1,\ldots ,m$, there are *c* edited attribute vectors $y_{j}^{s},s=1,\ldots ,c$. Therefore, we can get *c* pieces of evidence indicated by BBAs.

The BBA construction strategy is always designed based on the distance between the object $y_{j}$ and the training class $w_{g}$. This strategy should satisfy the following principles:

1. With an increase in this distance, the belief of $y_{j}$ belonging to class $w_{g}$ should have a monotone decrease.
2. The sum of all BBAs should equal 1.
3. The value of each BBA should be limited to the interval [0,1].

Considering the imperfection of the sensor’s observation, we used the interval of the upper and lower limits for the attribute values in the class $w_{g}$ to express the corresponding attribute of $w_{g}$. These attribute intervals are treated as interval numbers (INs). Thus, the attribute distances between the object $y_{j}$ and the training class $w_{s},s=1,\ldots ,c$ can be computed by the distances between their attribute INs.

Let us consider that the training samples have *k* dimensions. The interval number of the *v-*th dimension $w_{sv},v=1,\ldots ,k$ can be defined as:

(11) $$w_{sv}=[\min x_{iv},\max x_{iv}],x_{i}\in w_{s}.$$

At the same time, the interval number of the object $y_{j}$ on the *v-*th dimension is defined as $y_{jv}=\left[y_{jv},y_{jv}\right],v=1,\ldots ,k$. The distance between a pair of INs is introduced by Theorem 1.

**Theorem** **1.** *Let the distance between two INs* $A\left(a_{1},a_{2}\right)$ *and* $B\left(b_{1},b_{2}\right)$ *be defined as* *follows* [36]:

(12) $$\begin{array}{l} D^{2}\left(A,B\right)={\int_{-1/2}^{1/2}\left\{\left[\frac{\left(a_{1}+a_{2}\right)}{2}+x\left(a_{2}-a_{1}\right)\right]-\left[\frac{\left(b_{1}+b_{2}\right)}{2}+x\left(b_{2}-b_{1}\right)\right]\right\}}^{2}dx\\ ={\left[\frac{\left(a_{1}+a_{2}\right)}{2}-\frac{\left(b_{1}+b_{2}\right)}{2}\right]}^{2}+\frac{{\left[\left(a_{2}-a_{1}\right)+\left(b_{2}-b_{1}\right)\right]}^{2}}{12} \end{array}.$$

According to Theorem 1, the distance between $y_{jv}$ and $w_{sv}$ can be computed as follows:

(13) $$d_{jsv}=\sqrt{{\left[y_{jv}-\frac{\left(\min x_{iv}+\max x_{iv}\right)}{2}\right]}^{2}+\frac{{\left[\left(\max x_{iv}-\min x_{iv}\right)\right]}^{2}}{12}},v=1,\ldots ,k.$$

Therefore, the distance between the object $y_{j}=[y_{j1},\ldots ,y_{jk}]$ and $w_{s}=[w_{s1},\ldots ,y_{sk}]$ can be computed by their attribute distance.

In machine learning, there are many types of functions satisfying the principles of the BBA construction strategy. For convenience, we use exponential functions as the mapping function to convert the distances to BBAs. For edited attribute vectors $y_{j}^{s}$, the corresponding BBA on the singleton class $w_{s}$ and compound class Ω can be computed by:

(14) $$m_{j}^{s}(w_{s})=e^{-\lambda d_{js}}$$

(15) $$m_{j}^{s}(\Omega )=1-e^{-\lambda d_{js}}$$

with

(16) $$d_{js}=\frac{1}{k}\sum_{z=1}^{k}d_{jsv},v=1,\ldots ,k$$

where k is the number of attribute values in the object $y_{j}^{s}$, and 1/k is used to normalize the attribute distances. $d_{jsv}$ is the attribute distance on the *v-*th dimension between the object $y_{j}^{s}$ and the class $w_{s}$. λ is a tuning parameter that is used to manage the value of the BBA on the singleton class $w_{s}$. The parameter λ is suggested to be selected from 0.5 to 0.8 [6].

The generated BBA can be regarded as a piece of the classification result. In a *c*-class problem, we can get *c* edited attribute vectors by using ELMI. Thus, there are *c* pieces of classification results in relation to the object $y_{j}$.

#### 3.2.2. The Fusion of the Classification Results

The *c* possible estimations of each missing attribute are derived from different training classes $T_{s},s=1,\ldots ,c$. Consequently, the *c* piece of the classification results may conflict with the decision of the object class. If the classical Dempster’s combination rule is adopted directly to combine these classification results, then an unreasonable combined result will probably be generated. To avoid this problem, we use the scheme from [10] and transfer the conflicting BBAs to selected compound classes. The decision for each piece of the classification result corresponds to the class with maximum Bel(⋅). The classification results that have the same decision are divided into group $G_{p},p=1,\ldots ,q$. Thus, we can get q groups. The combined results in the same group are non-conflicting, as they have the same decision for the object class. For each group, Dempster’s combination rule is applicable for combining the classification results, and the combined results of different groups are called the sub-combined results.

In $G_{p}=\{m_{j}^{h},\ldots ,m_{j}^{f}\}$, the sub-combined results of the BBAs (classification results) calculated using Dempster’s combination rule are shown as follows:

(17) $$m_{j}^{w_{p}}\left(A\right)=\left[m_{j}^{h}⊕\ldots ⊕m_{j}^{k}\right]\left(A\right)$$

where ⊕ means the Dempster’s combination operation.

Considering that the classification results in different groups are conflicting, the sub-combined results of different groups are also mutually conflicting. Since Dempster’s combination rule is unavailable in such conflicting cases, the combination rule proposed by Liu et al. in [10] was adopted to fuse the sub-combined results derived from different groups.

Assume that the combined BBA of group $G_{p}$ strongly supports the class $w_{p}$ as its decision, i.e., $Bel_{j}^{w_{p}}\left(w_{p}\right)=\max\left(Bel_{j}^{w_{p}}\left(\cdot \right)\right)$. It means that the decision of $G_{p}$ supports class $w_{p}$.

Considering all possible classes of the object, the class $w_{\max}$ is the most supported by all sub-combined results if $w_{\max}$ satisfies the following condition:

(18) $$Bel_{j}^{w_{\max}}\left(w_{\max}\right)=\max\left(Bel_{j}^{w_{1}}\left(w_{1}\right),\ldots Bel_{j}^{w_{s}}\left(w_{s}\right)\right).$$

Liu et al. considered that $w_{\max}$ is the most possible class of the object. In addition, if there are other classes $w_{p}$, and the values of $Bel_{j}^{w_{p}}\left(w_{p}\right)$ are very close to the value of $Bel_{j}^{w_{\max}}\left(w_{\max}\right)$, then the object may also belong to $w_{p}$. As a result, the set of potential classes of the object is defined as follows:

(19) $$\Lambda_{j}=\left\{w_{p}|Bel_{j}^{w_{\max}}\left(w_{\max}\right)-Bel_{j}^{w_{p}}\left(w_{p}\right)<\varepsilon \right\}$$

where ε∈[0,1] is a threshold that is selected based on the cross-validation in the training data space. The classes of the set $\Lambda_{j}$ are not distinguishable. Any singleton element of the set $\Lambda_{j}$ is likely to be regarded as the real class of the monitoring object, and it is necessary to keep all the subsets of $\Lambda_{j}$ in the final combined result. Therefore, the combination rule for sub-combination results is defined as follows: $\forall B_{j}\subseteq \Omega$

(20) $$\tilde{m_{j}}\left(A\right)=\{\begin{array}{c} \mathrm{for}\;A\in \Omega \;\mathrm{with}\;\left|A\right|=1,\;or\;A=\Omega \\ \sum_{\overset{q}{\underset{s=1}{\cap }}B_{s}=A}m_{j}^{w_{1}}\left(B_{1}\right)\cdots m_{j}^{w_{q}}\left(B_{q}\right)\\ \mathrm{for}\;A\subseteq \Lambda_{j}\;\mathrm{with}\;\left|A\right|\geq 2\\ \sum_{\underset{\overset{\left|A\right|}{\underset{j=1}{\cup }}B_{j}=A}{\overset{\left|A\right|}{\underset{j=1}{\cap }}B_{j}=\phi }}\left[m_{j}^{w_{1}}\left(B_{1}\right)\cdots m_{j}^{w_{q}}\left(B_{q}\right)\overset{q}{\underset{s=\left|A\right|+1}{\Pi }}m_{j}^{w_{s}}\left(\Omega \right)\right] \end{array}$$

where q is the number of groups, and |A| is the cardinality of A. Due to some singleton classes not being included in set $\Lambda_{j}$, the final combined BBA should correspond to the normalized $\tilde{m_{j}}\left(A\right)$, formally defined by:

(21) $$m_{j}\left(A\right)=\frac{\tilde{m_{j}}\left(A\right)}{\sum_{B_{i}}\tilde{m_{j}}\left(B_{i}\right)}.$$

The final decision is made based on the final combined BBA directly. According to the credal classification method, the object can be assigned a singleton class or a compound class with the maximum value of the final combined BBA. The compound class is used here to reveal the imprecision of the data caused by missing values. If the object is classified into a singleton class, it reflects that our proposed method is appropriate for the classification of incomplete data. The ELMI can provide rational estimations for the missing values. If the object is classified into a compound class, then the object probably belongs to any singleton class included in set $\Lambda_{j}$, but we cannot make a specific decision according to the known values. It also reveals that the missing values are crucial for precise classification.

Algorithm 1 describes the pseudo-code of the proposed method.

**Algorithm 1**. The combination of incomplete data.**Input:** Training data: $X=\{x_{1},\ldots ,x_{n}\}$.   Test data: $Y=\{y_{1},\ldots ,y_{m}\}$.Parameters: ε∈[0,1]: threshold for compound class.  for *j* = 1 to *m*  Calculate *c* versions of estimations of the missing attributes by ELMI.  Classify *c* edited attribute vectors $y_{j}$ with missing values by Equations (14) and (15).  Sub-combination of classification results in the same group using Equation (17).  Select compound classes by Equation (19).  Combination of the sub-combination results using Equations (20) and (21).  end

## 4. Experimental Results

In this section, two experiments are carried out to evaluate the performance of our proposed classification method for incomplete data. For performance comparison, three other popular classification approaches based on the MI, KNNI, and SVRI will be used. In MI, the missing attribute values are filled with the mean values of the same attributes of the training data. In KNNI, the missing attribute values are filled with the values of the same attributes of its K-nearest neighbors. In SVRI, the missing values are estimated by minimizing the combination of the training error and a regularization term. Because the ELM has the better performance with a fast learning speed [34], the test data with estimated values are classified using the regularized ELM in this work, and all the objects are classified into the singleton class. In the classification with our proposed method, the decision on the object class is made according to the class with the maximum value of the final combined BBA. Thus, each object can be classified into a singleton class or a compound class. To evaluate the performance of classification, the following measures proposed in [9] are used in our experiments:

1. The misclassification declared for the object corresponds to $w_{i}$ if it is classified into A with $w_{i}\cap A=\phi$.
2. If $w_{i}\cap A\neq \phi$ and $w_{i}\neq A$, it will be regarded as an imprecise classification.
3. The error rate is denoted by Re=Ne/T, where $N_{e}$ is the number of misclassification errors, and T is the number of test objects.
4. The imprecise rate is denoted by Ri=Ni/T, where Ni is the number of objects classified into compound classes.

### 4.1. Experiment on Transformer Fault Diagnosis

In this experiment, we use the data collected from gas sensors to detect the types of states of the transformer [3]. There are many kinds of gases in the transformer’s inner space, such as H_2_, CH_4_, C_2_H_6_, and C_2_H_4_. When different faults occur, the percentage of each gas will change. Considering that a transformer has three types of states, including the discharge fault state (Di), temperature fault state (Te), and normal state (No), we use C_2_H_6_ and C_2_H_4_ to diagnose the state of a transformer.

**Figure 3:**
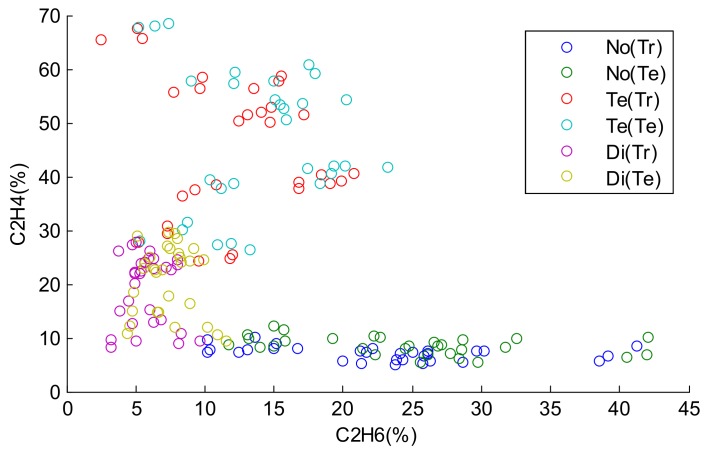
The distributions of the training samples and test samples.

In this work, there are 60 samples in each state. The frame of discernment is Ω={No, Di, Te}. In order to evaluate the performance of our proposed method, 30 samples of each state are selected as the training samples, and the remaining 30 samples are used as the test samples. The distributions of the training samples and test samples are shown in Figure 3.

In Figure 3, No(Tr), Te(Tr), and Di(Tr) represent the training samples of the normal state (No), temperature fault state (Te), and discharge fault state (Di), respectively. No(Te), Te(Te), and Di(Te) represent the test samples of the normal state (No), temperature fault state (Te), and discharge fault state (Di), respectively. The horizontal axis and vertical axis denote the percentage of C_2_H_6_ and C_2_H_4_, respectively. From Figure 3, we can see that the lower bound of Te and the upper bound of Di are intersecting, and the lower bound of Di and the upper bound of No are intersecting. In these cases, if the values in the horizontal axis are missing, it is hard to classify the samples in the intersection scope into their correct states. Consequently, we assume that the values in the first dimension corresponding to the horizontal axis of the test samples are missing, and the classification of the test samples depends on the values in the vertical axis.

**Figure 4:**
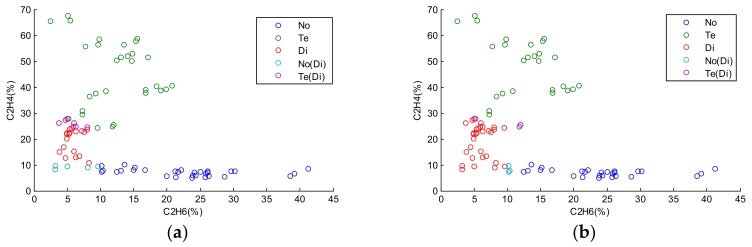
Classification results by different approaches: (**a**) Classification result by regularized ELM with mean imputation (MI) (Re=12.2%); (**b**) Classification result by regularized ELM with K-nearest neighbor imputation (KNNI) (Re=6.67%).

In Figure 4, the missing values of test samples in the horizontal axis are estimated by KNN and the mean values of the training samples, respectively, and then the test samples with the estimated values are classified by the regularized ELM. The blue circles represent the misclassification errors between No and Di. The purple circles represent misclassification errors between Te and Di. Both of these two approaches have high error rates. The reason is that both MI and KNNI provide only one estimation. The test samples in the intersection scope can be classified into different states with their corresponding estimations, and one estimation cannot reflect such uncertainty and imprecision resulting from missing values.

**Figure 5:**
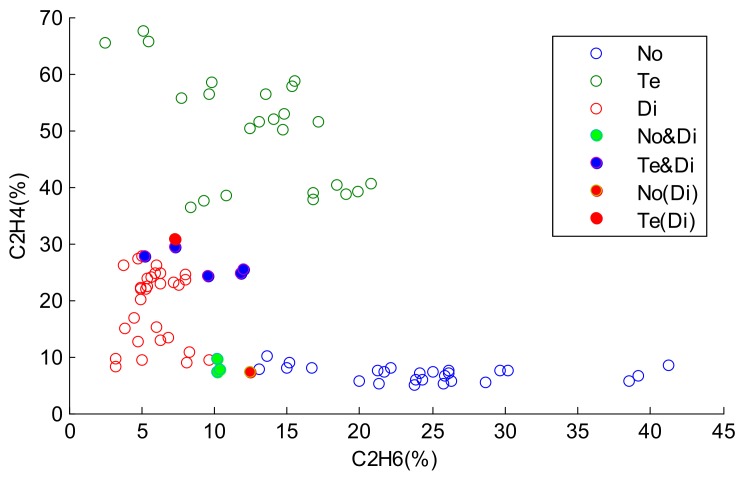
Classification result by our proposed method (Re=2.22%,Ri=8.89%).

As shown in Figure 5, there are two samples with misclassification errors and eight samples classified into compound classes. This show that our proposed method has a better performance than the other two approaches. Most samples with missing values in the intersection scope are classified into appropriate compound classes. This reveals the uncertainty and imprecision caused by missing values. The multiple possible estimations provided by ELMI are sufficient to reduce the misclassification errors with a reasonable imprecision rate.

### 4.2. Experiment on Machine Learning

In this experiment, six data sets (the breast cancer, iris, seeds, segment, satimage, and wine data sets) from the University of California Irvine (UCI) machine learning repository are chosen to evaluate the performance of our proposed method. The specifications of the selected data sets are given in Table 1.

We use the fivefold cross-validation method to test different classification methods on the six data sets [37]. It is assumed that each test sample has *n* missing values at random in every dimension. The popular Sigmoid additive hidden node is used in regularized ELM. According to [16], a good performance of ELM with the Sigmoid additive node can be obtained as long as the number of hidden nodes is large enough. Thus, the hidden nodes number is set as 1000 in this work. The average error rate Re and imprecision rate Ri are shown in Table 2.

As observed from Table 2, SVRI and ELMI have better performances than the other methods. The classification based on ELMI produces a lower average error rate Re than the classification based on SVRI in most cases. This is because some undistinguishable samples with missing values are classified into compound classes, which leads to some imprecise classification results. As the number (*n*) of missing values in each test sample increases, the error rate and imprecision increase as well. This reflects that more missing values can cause higher uncertainty, which makes the objects hard to classify into the singleton classes, and it also reveals that the missing values play a crucial rule in the classification process. Compared with other approaches, our classification method designed based on ELMI and evidential reasoning can rationally deal with the uncertainty caused by missing values, and classifies the incomplete samples into proper compound classes. As a result, the performance of the proposed method is better than that of the other two traditional methods.

For the purpose of evaluating the efficiency of ELMI, the training time and testing time for all data sets are given in Table 3. Considering that the classification method with KNNI needs to compute the distances between the object and every training sample, it imposes a huge computation burden. Furthermore, MI always offers imprecise results in most cases. As a result, SVRI and ELMI, which have better performances in error rate, are compared in their average running time. The training time includes the training time of both imputation and classification, and the testing time includes the testing time of both imputation and classification.

Obviously, the ELMI method provides a much better performance than SVRI method in the running time for the selected data sets. Meanwhile, ELMI usually affords the lowest error rate in these approaches. Thus, considering the limitations of the energy and calculation capacity of sensor nodes, the proposed method can be an efficient classification method to deal with incomplete data in WSNs.

## 5. Conclusions

The classification of incomplete data with missing attribute values is a common problem in WSNs. Limited by the energy and calculation capacity of the sensor nodes, complicated classification methods are not suitable for WSNs. Consequently, a novel classification method of incomplete data based on evidential reasoning and ELM is proposed in this paper. This method uses the simple and efficient regularized ELM to estimate the missing values of incomplete data. The regularized ELM can provide multiple estimations, and thus all potential values for incomplete data are estimated. Then, the complete data with the estimated values is converted into BBAs by a new BBA construction strategy, which employs interval numbers to calculate the distance between an object and the training class. Lastly, a flexible evidence combination rule is adopted to fuse the generated multiple BBAs, where the undistinguished classes caused by missing values are selected as compound classes. In this way, our proposed method realizes a good balance between error and imprecision. The experimental results show that this method has a better performance than other traditional classification methods for incomplete data. In our future work, we attempt to improve our scheme in the following respects: (1) A simpler combination rule is needed to realize a good balance between error and imprecision; and (2) We will try to find another way to estimate the missing values of the incomplete data.

## Figures and Tables

**Table 1:** The specification the selected data sets.

Name	Classes	Attributes	Instances
Breast	2	9	699
Iris	3	4	150
Seeds	3	7	210
Wine	3	13	178
Segment	7	19	2310
Satimage	6	36	6435

**Table 2:** The classification results for different data sets.

Data set	*n*	MI Re	KNNI Re	SVRI Re	ELMI {Re, Ri}
Breast	3	4.79%	4.86%	4.71%	{4.52%, 3.18%}
	5	8.23%	8.38%	5.96%	{4.90%, 3.31%}
	7	14.75%	14.29%	12.45%	{10.42%, 5.59%}
Iris	1	23.96%	5.65%	5.13%	{4.77%, 2.09%}
	2	43.67%	13.72%	8.07%	{8.12%, 6.52%}
	3	65.49%	21.06%	12.89%	{12.92%, 9.23%}
Seeds	2	21.05%	12.38%	9.26%	{9.47%, 4.13%}
	4	28.39%	13.62%	10.83%	{9.65%, 4.86%}
	6	40.93%	27.34%	18.37%	{17.02%, 12.53%}
Wine	3	31.26%	27.18%	26.51%	{26.17%, 1.64%}
	6	33.98%	27.93%	27.14%	{26.83%, 1.58%}
	9	38.02%	30.36%	28.33%	{27.25%, 3.67%}
Segment	3	12.71%	10.46%	9.52%	{6.89%, 1.37%}
	7	15.83%	12.38%	10.07%	{7.16%, 3.25%}
	11	20.23%	17.35%	12.06%	{10.35%, 3.76%}
Satimag	7	41.28%	40.70%	33.62%	{29.63%, 12.81%}
	9	44.75%	42.36%	36.59%	{30.87%, 18.24%}
	19	52.96%	51.22%	47.67%	{36.92%, 22.75%}

SVRI: support vector regression imputation.

**Table 3:** The comparison of training and testing time of SVRI and ELMI.

Data Set	SVRI		ELMI	
	Training	Testing	Training	Testing
Breast	3.8371 s	0.9353 s	0.9761 s	0.2572 s
Iris	1.0639 s	0.2108 s	0.1924 s	0.0497 s
Seeds	2.4271 s	0.5141 s	0.4212 s	0.1644 s
Wine	2.1533 s	0.5279 s	0.3125 s	0.1398 s
Segment	753.2 s	1.0422 s	2.32 s	0.5461 s
Satimag	2157.3 s	2.1823 s	15.79 s	0.5633 s
